# On-Chip Cryopreservation: A Novel Method for Ultra-Rapid Cryoprotectant-Free Cryopreservation of Small Amounts of Human Spermatozoa

**DOI:** 10.1371/journal.pone.0061593

**Published:** 2013-04-30

**Authors:** Yujie Zou, Tailang Yin, Shijing Chen, Jing Yang, Weihua Huang

**Affiliations:** 1 Reproductive Medicine Center, Renmin Hospital of Wuhan University, Wuhan, China; 2 Center of Reproductive Medicine, Department of Obstetrics and Gynecology, Peking University Third Hospital, HaiDian District, Beijing, China; 3 Key Laboratory of Analytical Chemistry for Biology and Medicine (Ministry of Education), College of Chemistry and Molecular Sciences, Wuhan University, Wuhan, China; University of Nevada School of Medicine, United States of America

## Abstract

Cryopreservation of human spermatozoa free from cryoprotectant can avoid toxicity caused by highly concentrated cryoprotectant and a series of specific carriers have been previously explored, except for PDMS chip. Our study is aimed at exploring a novel device for ultra-rapid cryopreservation of small numbers of spermatozoa without cryoprotectant based on polydimethylsiloxane (PDMS) chips. Spermatozoa from 25 healthy men were involved in this study, comparing on-chip cryopreservation with different micro-channel height (group A: 10 µm height, group B: 50 µm height, group C: 100 µm height) and conventional freezing (group D) in liquid nitrogen for 72 h. The viability, motility, DNA integrity by comet assay and acrosome integrity by fluorescein isothiocyanate-conjugated peanut agglutinin (FITC-PNA) staining of frozen-thawed spermatozoa of each group were compared. The motility and viability of post-thawed spermatozoa was significantly decreased than that of pre-freezing spermatozoa. There was no difference of viability and motility of frozen-thawed spermatozoa between group A and D, while viability and motility of group B and C decreased compared to group A. Comet assay showed that no matter for group A or D, there was no difference of CR, TL, TD and OTM between pre-frozen and post-thawed spermatozoa. There was no difference of CR, TL, TD and OTM of post-thawed spermatozoa between group A and group D neither, while spermatozoa DNA damage was more serious in group B and group C with increasing height of micro-channel compared with group A. The proportion of intact acrosome of post-thawed spermatozoa in group A was the highest when compared with group B and group C, though similar to that of group D. In conclusion, PDMS chip with 10 µm height micro-channel is ideal for ultra-rapid cryopreservation of small quantity of spermatozoa without cryoprotectant.

## Introduction

Male fertility has declined during the past several decades and the number of patients with azoospermia and oligospermia is increasing [1]. Testicular spermatozoa extraction (TESE) is the common way to obtain spermatozoa from patients with azoospermia, which is expensive, time-consuming and destructive to blood-testicular barrier. In particular, the patients have to bear the repeated surgery procedure after each ICSI failure. Thus, it's necessary to find a method for effective cryopreservation of small amounts of human spermatozoa.

Microfluidic technology is an emerging subject manipulating miniaturized fluids based on chips with micro-fabricated channels and chambers. Microfluidic technology has been utilized in numerous biological applications specifically for miniaturization and simplification of laboratory techniques [2]. Polydimethylsiloxane (PDMS) is the commonest material for making a chip, and it has been proved to be safe and friendly to reproductive cells [3]. Here we just develop a novel method for ultra-rapid cryopreservation of small amounts of human spermatozoa without cryoprotectant using a PDMS chip for the first time.

## Materials and Methods

### Device design and fabrication

**Figure 1:**
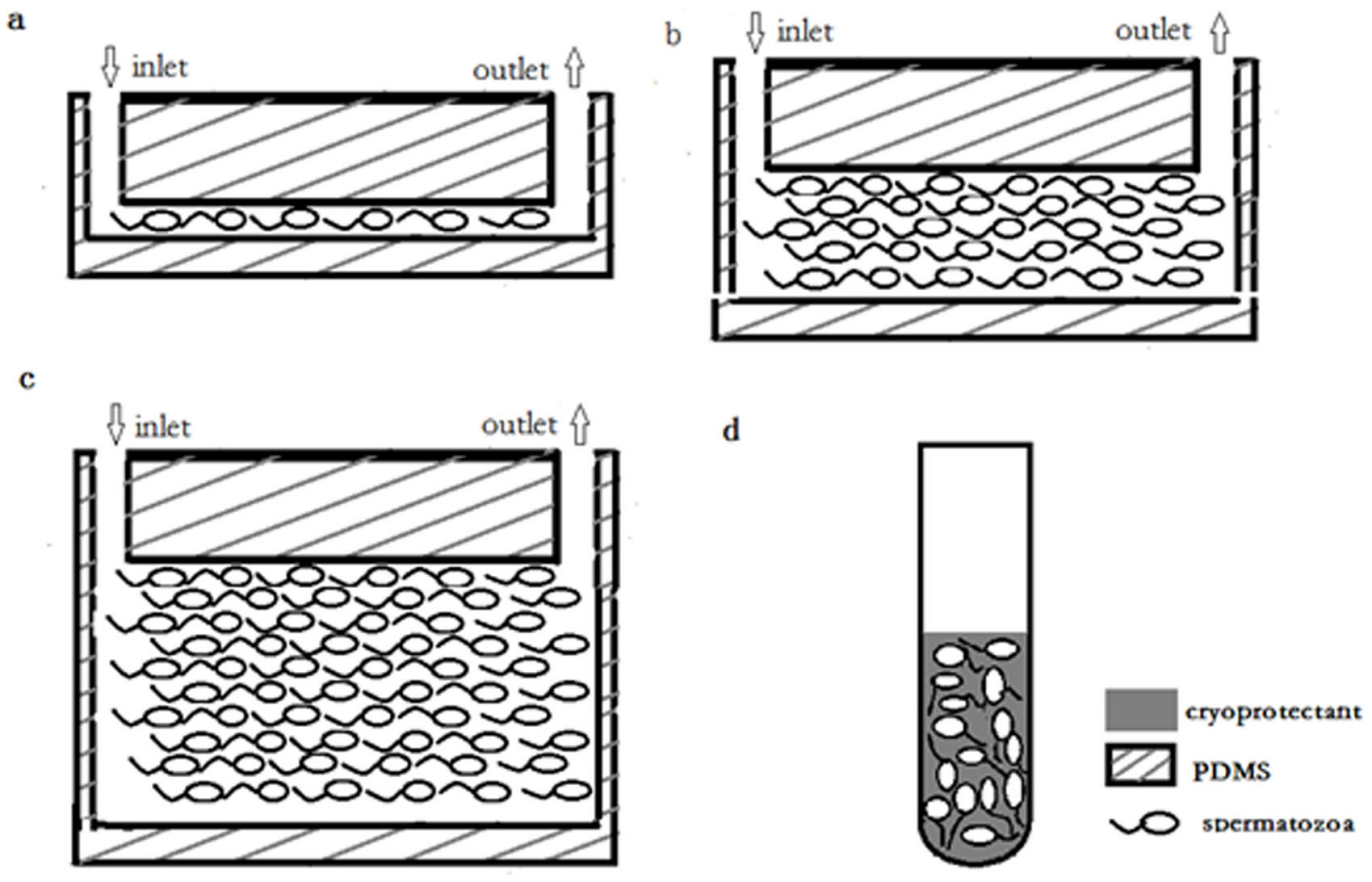
Schema of spermatozoa store in PDMS chip and freezing tube. **a.** spermatozoa cryopreserved in micro-channel of which height is 10 µm (group A) one by one; **b.** spermatozoa cryopreserved in micro-channel of which height is 50 µm (group B); **c.** spermatozoa cryopreserved in micro-channel of which height is 100 µm (group C); **d.** spermatozoa cryopreserved disorderly in a 1.8 ml freezing tube (group D).

**Figure 2:**
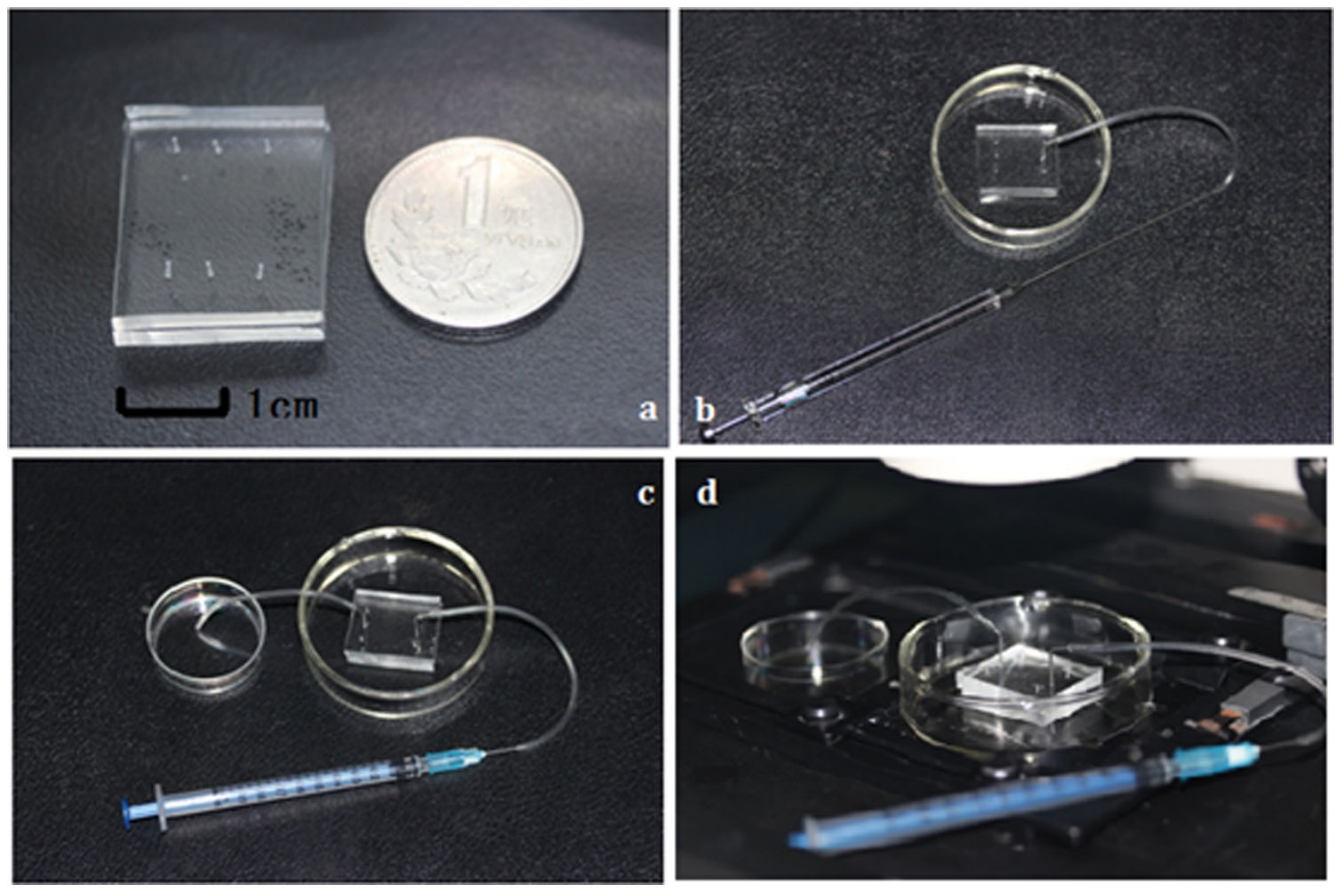
Workflow of PDMS chip. **a.** a PDMS chip compared with a coin; **b.** sample loading with a micro-injector; **c.** thawing of frozen spermatozoa; **d.** push the syringe and thawed spermatozoa in micro-channel is observed to be transferred into the tissue culture dish under the microscope.

We strictly obeyed the Declaration of Helsinki for Medical Research involving Human Subjects during the project and written consent was obtained from all subjects. This study was approved by the Ethics Committee of Renmin Hospital, Wuhan University. As shown in Figure 1a, 1b and 1c, the chip was comprised of three regions: spermatozoa inlet, spermatozoa store channel and spermatozoa outlet. To find the best parameter for channel design, we designed three channels of different heights: 10 µm, 50 µm and 100 µm. The width and length of the three channels were separately 10 µm and 5 cm. The volume of 10 µm, 50 µm and 100 µm height channel was separately 5×10^−3^ µL, 25×10^−3^ µL and 50×10^−3^ µL. The chip was fabricated with two layers of PDMS. Figure 2a showed the chip compared with a coin. The size of the chip was about 4 cm (length)×3 cm (width)×0.5 cm (height). The upper layer had a micro-channel with an inlet and an outlet punched separately at each ends. The lower layer was smooth without any structures at the surface and plasma bonded together with the upper layer to hold the whole system.

### Validation of the method

A chip was stored in the liquid nitrogen for 72 h, and then taken out and recovered in 37°C water bath for 10 min. To detect the tolerance of PDMS to very low temperature in liquid nitrogen, we examined the planeness of surface and elasticity of whole device. Then we loaded some deionized water with a syringe into the channel of chip, and observed whether the channel became deformed or the fluid went forward without any difficulty in the channel.

### Collection of spermatozoa sample

Ejaculates were obtained from 25 healthy men by masturbation after at least 72 h of sexual abstinence. 12 samples were used for detection of spermatozoa motility, viability and DNA integrity, and 13 samples were used for acrosome integrity evaluation. The ejaculates were required to contain 20 million or more spermatozoa/mL and to show at least 50% progressive spermatozoa motility and 14% morphologically normal spermatozoa. Informed consent was obtained from each donor. Semen analysis was performed according to published guidelines of the World Health Organization [4].

### PureSperm gradient centrifugation

The PureSperm gradient centrifugation was performed according to the PureCeption™ Sperm Separation Media protocol. To prepare the single bilayered gradient for spermatozoa purification, 2 mL of Lower Phase (PureCeption™ 80%) was pipetted into the bottom of the centrifuge tube and 2 mL of Upper Phase (PureCeption™ 40%) was carefully layered over the Lower Phase. 2.5 mL of liquefied semen was gently placed onto the Upper Phase of the single bi-layered gradient and centrifuged at 350 g for 20 min. Remove all layers except the lowest portion (approximately 0.3 mL), add 2 mL of Spermatozoa Washing Medium(PureCeption™, SAGE,USA, 5 mg/ml HEPES-buffered Human Tubal fluid with Human Serum Albumin) and resuspend the pellet. The resulting pellet, placed in the bottom of the tube, was centrifuged at 250 g for 6 min, aspirated and diluted in 0.5 mL Spermatozoa Washing Medium. After estimate of motility, viability, DNA integrity and acrosome integrity, the sample was divided into four equal parts for on-chip cryopreservation (group A: 10 µm; group B: 50 µm; group C: 100 µm) and conventional slow freezing (group D).

### On-chip cryopreservation and conventional freezing of human spermatozoa

**Figure 3:**
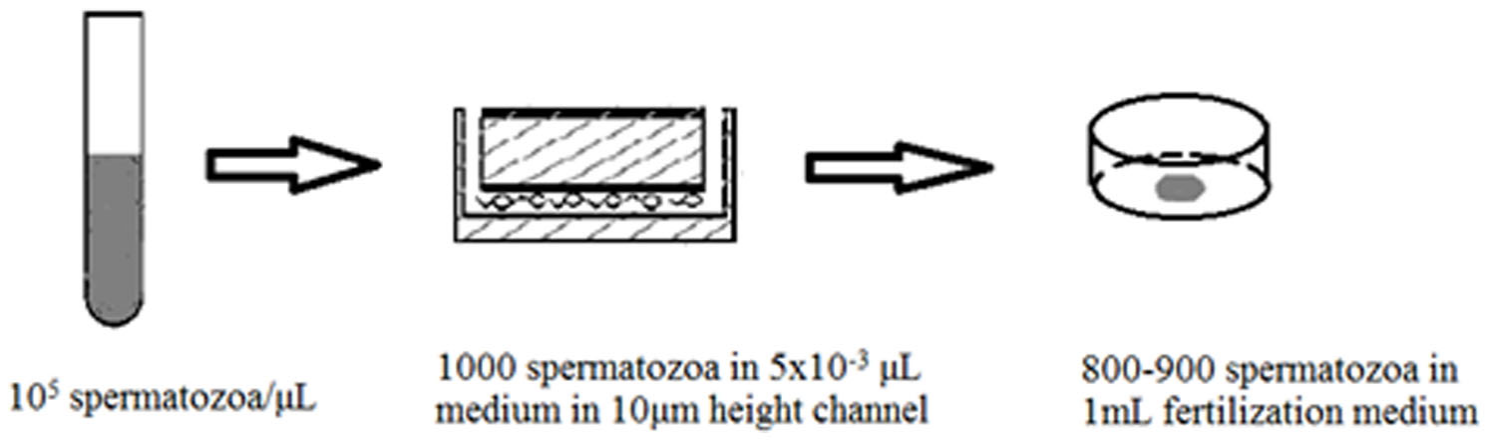
Schematic illustration of the volume distribution of sperm suspension in each step when cryopreserved in 10 µm height channel. The density of spermatozoa sample was adjusted to 10^5^/µL before loading. 5×10^−3^ µL medium containing 1000 spermatozoa can be stored in 10 µm height channel and 800–900 spermatozoa can be finally transferred out in 1 mL fertilization medium.

Before use, the chip was sterilized in 75% ethanol for 30 min, dried, and illuminated under UV for 30 min. Then the sample contacting surfaces of the chip were hydrophilized by plasma treatment. Before on-chip cryopreservation of group A, B and C, the density of spermatozoa sample was adjusted to 10^5^ cells/µL so that we can guarantee a high number of spermatozoa stored in the micro-channel. Then the spermatozoa was transferred into a soft tube connected with a needle by pulling a 5 µL micro-injector (Gaoge Industry Trade Co., Ltd., Shanghai, China) (Figure 2b). Next we inserted the needle into the inlet of chip and pushed the micro-injector slowly so that the spermatozoa were administrated into the channel (Figure 2c). Take the 10 µm height channel for example, 5×10^−3^ µL washing medium containing 1000 spermatozoa was thus stored in the micro-channel (Figure 3). For 50 µm and 100 µm height channel, the volume distribution in micro-channel just increased by 5 folds and 10 folds. All the performance was observed under a microscope at the same time, so that we could stop pushing the syringe when the whole channel was filled with spermatozoa (Figure 2d). Wrap the whole chip with silver paper as a protective layer from direct contact with liquid nitrogen. Keep it in the liquid nitrogen for 72 h. Conventional freezing of group D: Spermatozoa sample was mixed with cryoprotectant (Quinn's Advantage@ spermatozoa Freezing Medium, SAGE,USA) of equal volume size and the mixture was allowed to equilibrate for 3 minutes at room temperature. Quinn's Advantage@ spermatozoa Freezing Medium was a HEPES-buffered salt solution containing 10 mg/mL human serum albumin, glycerol and sucrose as the cryoprotective agent, and gentamicin as an antibiotic. The 1.8 mL freezing tube (Corning Costar, USA) was first placed at 4°C for 60 min, and then transferred quickly to liquid nitrogen vapor (−80°C) which was about 10 cm above the surface of the liquid nitrogen for 30 min. Finally we quickly plunged the freezing tube into liquid nitrogen (−196°C) and stored for 72 h.

### Thawing procedure of frozen spermatozoa

Group A, B, C: Take the frozen chip out of liquid nitrogen, remove silver paper and recover in 37°C incubator for 10 min. After the spermatozoa were completely thawed, we slowly pushed the syringe connected to the inlet and observed until the spermatozoa were completely transferred into the tissue culture dish (BD FALCON, USA) under the soft tube connected to outlet. Take 10 µm micro-channel for example, about 800–900 out of 1000 spermatozoa could be finally transferred out in 1 mL fertilization medium(Quinn's Advantage@ Fertilization HTF Universal Medium,SAGE,USA) during this process. Group D: Take the freezing tube out of liquid nitrogen and warm in 37°C water bath for 10 min. After centrifugating at 400 g for 5 min, the pellet was collected and resuspended in 100 µL fertilization medium.

### Viability of frozen-thawed spermatozoa by eosin Y staining

Spermatozoa viability was assessed using eosin Y staining. The viability of cryopreserved spermatozoa in each group was determined before freezing and after 10 min thawing by 37°C incubation in group A, B and C or melting in 37°C water bath in group D. Considering a low density of post-thawed spermatozoa in group A, B and C, we directly used the 0.5 mL centrifugate tube instead of tissue culture dish to hold the thawed spermatozoa. After centrifugating at 400 g for 5 min, we collected and resuspended the spermatozoa in 5 µL fertilization medium. Then 2 µL drop of stain and 2 µL spermatozoa was placed on microscope slide. A coverslip was applied and the slide was examined with a 400× lens. The slides were scored for living and dead spermatozoa under light microscope (Olympus). Intact spermatozoa excluded the eosin and appeared white, while dead spermatozoa with damaged membrane appeared red. A minimum of 200 spermatozoa under a magnification of 400× was analyzed from each specimen.

### Motility of frozen-thawed spermatozoa

The motility of pre-frozen spermatozoa was assessed immediately after PureSperm gradient centrifugation. The motility of cryopreserved spermatozoa in each group was determined immediately after instant thawing by melting in 37°C water bath in group D or 37°C incubation in group A, B and C as described above. A Makler Counting Chamber was used for motility scoring. This index was estimated under the light microscope at a magnification of 400×. Only spermatozoa showing progressive motility were assessed.

### Assessment of spermatozoa DNA integrity by comet assay

We performed the comet assay using OxiSelect™ Comet Assay Kit. 1× Lysis Buffer(containing 3.65 mol/L NaCl, 50 mM/LEDTA), Alkaline Solution(300 mM/L NaOH, 1 mM/L EDTA), Electrophoresis Running Solution (300 mM NaOH, 1 mM EDTA, pH>13) were prepared following the protocol. Post-thawed spermatozoa was washed by centrifugation at 700 g for 2 min, and the pellet was washed with ice-cold PBS(without Mg^2+^ and Ca^2+^) giving a final concentration at 1×10^5^ cells/mL. 10 µL cell sample was mixed with 100 µL OxiSelect™ Comet Agarose and 75 µL/well was immediately pipetted onto the OxiSelect™ Comet Slide. The slide was maintained horizontally at 4°C for 15 min, then immersed in the pre-chilled Lysis Buffer (25 mL/slide) for 30 minutes at 4°C, and finally in the pre-chilled Alkaline Solution (25 mL/slide) for 30 min at 4°C. The above procedures were all performed in the dark. After draining off the fluid, slides were placed in a horizontal electrophoresis unit and were equilibrated in cold Alkaline Electrophoresis Solution for 20 min before being electrophoresed at 15 V (1 volt/cm) and 300 mA for 30 min at room temperature. After electrophoresis, the slides were placed in 70% ethanol for 5 min, air-dried and subsequently stained with 100 µL/well of 1× Vista Green DNA Dye for 15 min at room temperature. View slides by epifluorescence microscopy (Olympus) using a FITC filter at 400× magnification. Images were analyzed by Comet Assay Software (Comet Assay Internet Group, http://www.casp.of.pl). For each sample, a minimum of 100 cells were simultaneously scored for comet rate (CR, %), tail length (TL, length of the comet tail measured from head area to end of tail, µm), tail DNA percentage (TD, percent of DNA in the comet tail) and Oliver tail moment (OTM, tail DNA%× tail length).

### Assessment of acrosome integrity by FITC-PNA staining method

Acrosome integrity was evaluated using FITC-PNA (fluorescein isothiocyanate-conjugated peanut agglutinin) staining method, following the protocol of Sperm Morphology FITC-PNA staining kit (GENMED SCIENTIFICS. Shanghai, China) specific to stain the acrosome. The spermatozoa sample was centrifugated at 600 g for 5 min, collected and resuspended in BWW medium adjusting to a final concentration of 1×10^5^ cells/mL. 10 µL spermatozoa sample was smeared on a microscope slide, air dried, and then fixed in ice–cold methanol for 2 min. After drying of the fixed smears, 200 µL FITC-PNA stain (25 µg/mL) was gently spread over the smear and incubate in the dark for 30 min at room temperature. The slides were then rinsed with 200 µL of phosphate–buffered saline (PBS) solution to remove excess stain. The acrosome status of spermatozoa was monitored and photographed with an epifluorescence microscope (Olympus) using an excitation wavelength of 480 nm and emissions of 530 nm. According to Reid [5], acrosomes were classified as I = intact, II and III are intermediate forms, IV = reacted. The percentage of fluorescent acrosome-intact spermatozoa (I) was counted in at least 200 sperm cells under the fluorescence microscope at 1000× magnification.

### Statistical analysis

Results are presented as mean values ± SD. To identify significant differences, two groups were compared by the independent t-test using SPSS 11.0. Differences were considered statistically significant at P<0.05.

## Results

### Validation of the method

After recovery after cryopreservation in the liquid nitrogen for 72 h, we found that the surface of PDMS was still smooth and whole chip was elastic. After we loaded deionized water with a syringe into the channel of the chip, no deformation of the channel was detected and the fluid went forward without any difficulty in the channel. Thus, PDMS was believed to tolerate very low temperature in liquid nitrogen and the whole device can work well after thawing.

### Motility and viability of frozen-thawed spermatozoa

As shown in Table 1, the motility and viability of thawed spermatozoa was significantly decreased than that of pre-freezing spermatozoa. There is no significant difference of motility and viability between group A and group D, while the motility and viability of group B and group C is significantly lower than that of group A. Namely, the wider the channel is, the lower viability and motility could be.

**Table 1:** Comparison of viability and motility of frozen-thawed spermatozoa among the groups.

Group	Number	Pre-freezing		Post-thawing	
		viability(%)	motility(%)	viability(%)	motility(%)
A(10 µm height)	12	88.6±3.0^a^	87.1±3.1^a^	51.3±2.8	49.5±2.8
B (50 µm height)	12	88.6±3.0^a^	87.1±3.1^a^	34.2±2.5^b^	33.5±2.6^b^
C (100 µm height)	12	88.6±3.0^a^	87.1±3.1^a^	13.2±2.0^b^	12.1±2.1^b^
D(conventional freezing)	12	88.6±3.0^a^	87.1±3.1^a^	50.9±2.2	49.1±2.5

a indicates a statistical difference when compared with post-thawing spermatozoa from Group A to Group D (P<0.05);

b indicates a statistical difference when compared with Group A (P<0.05).

### Assessment of spermatozoa DNA integrity by comet assay

**Figure 4:**
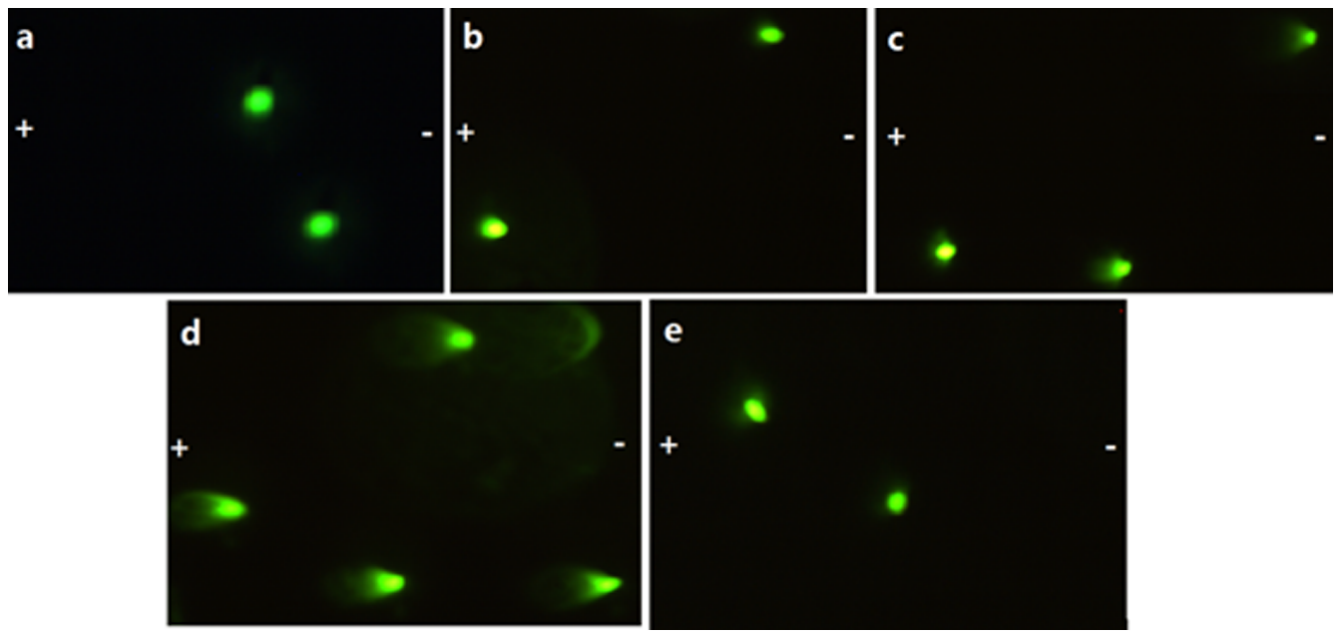
Photographs showing DNA integrity of frozen-thawed spermatozoa in each group at a magnification of 400×. The symbol “−” and “+” represent cathode and anode respectively during electrophoresis of negatively charged DNA. **a.** pre-frozen spermatozoa; **b.** post-thawed spermatozoa of Group A; **c.** comet tail of post-thawed spermatozoa in group B; **d.** obvious long comet tail in post-thawed spermatozoa of Group C; **e.** post-thawed spermatozoa of Group D.

Spermatozoa with undamaged DNA did not form a ‘comet’ and was considered as a viable sperm (Figure 4a). Sperms with low (Figure 4c) and high (Figure 4d) degree of DNA damage due to frozen–thawed process displayed increasing migration of the DNA from the nucleus towards the anode. No matter for group A or D, there was no difference of CR, TL, TD and OTM between pre-frozen and post-thawed spermatozoa. There was no difference of CR, TL, TD and OTM of post-thawed spermatozoa between group A and D neither. Compared with group A, spermatozoa DNA damage was more serious in group B and group C with increasing height of micro-channel (Table 2).

**Table 2:** Comparison of DNA integrity of frozen-thawed spermatozoa among groups.

Group		Number	CR(%)	TL(µm)	TD(%)	OTM
Pre-freezing		12	11.4±2.9	15.2±2.6	25.9±8.0	3.91±1.0
Post-thawing	A(10 µm height)	12	12.3±2.6	16.0±2.2	26.6±7.4	4.19±0.7
	B(50 µm height)	12	22.8±3.0^a^	19.5±3.2^a^	27.4±4.9	5.35±0.9^a^
	C(100 µm height)	12	45.8±3.1^a^	23.6±5.3^a^	33.0±5.2^a^	7.39±0.7^a^
	D(conventional freezing)	12	12.8±2.2	16.9±3.4	27.8±7.6	4.46±1.0

a indicates a statistical difference when compared with Group A (P<0.05).

### Assessment of acrosome integrity by FITC-PNA staining

**Figure 5:**
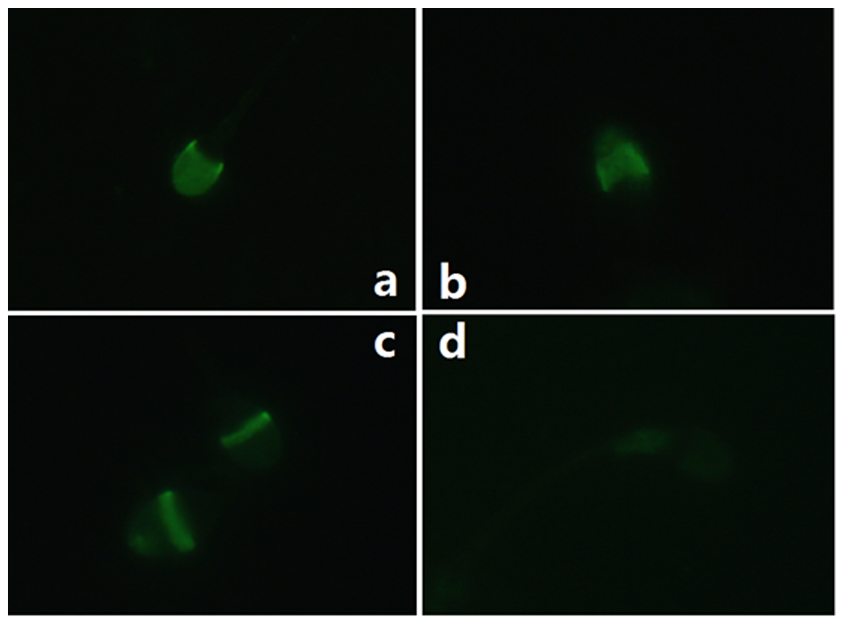
Photographs of four categories of acrosome status evaluated by FITC-PNA staining at a magnification of 1000×. **a.** I-intact acrosome; **b.** II-intermediate form of minimal acrosome reactivity; **c.** III-intermediate form of severe acrosome reactivity; **d.** IV-reacted acrosome.

The acrosome status of frozen-thawed spermatozoa among the groups was evaluated by FITC-PNA staining. Human spermatozoa stained with FITC-PNA (Figure 5) were classified into four catergories: I (Figure 5a), spermatozoa displaying intensively bright fluorescence of the acrosomal cap, indicating an intact acrosome; II (Figure 5b) and III (Figure 5c) are intermediate forms, displaying disrupted fluorescence of the acrosomal cap or fluorescence only shown in equatorial plane of spermatozoa respectively; IV(Figure 5d), spermatozoa displaying little or no fluorescence, indicating reacted acrosome. As shown in Table 3, the proportion of intact acrosome of post-thawed spermatozoa in group A was 72.8±3.6, while in group B and C, the proportion of intact acrosome was markedly reduced to 61.1±4.0 and 45.3±3.1 respectively. However, proportion of intact acrosome of post-thawed spermatozoa in group A showed no difference when compared with that of spermatozoa in group D (72.8±3.6 vs. 72.6±3.2), indicating a similar acrosome integrity between on-chip cryopreservation in 10 µm height channel and conventional freezing method.

**Table 3:** Comparison of acrosome integrity of frozen-thawed spermatozoa among groups.

Group		Number	Intact acrosome(%)
Pre-freezing		13	75.4±3.9
Post-thawing	A(10 µm height)	13	72.8±3.6
	B(50 µm height)	13	61.1±4.0^a^
	C(100 µm height)	13	45.3±3.1^a^
	D(conventional freezing)	13	72.6±3.2

a indicates a statistical difference when compared with Group A (P<0.05).

## Discussion

There are two potentially fatal mechanisms during the cryopreservation process, excessive osmosis and intracellular ice damages. Ice formation occur firstly in the extracellular aqueous solution during the freezing process and results in high solute concentration surrounding the living cells, which causes the osmosis effect to drive water out of the living cells. If the cooling rate is too slow, osmosis can remove water from cells excessively, leading to a harmful high electrolyte concentration inside cells. If the cooling rate is too fast, intracellular ice could form and become the recrystallization sites of large ice crystals later during the thawing process and could damage the cell membrane [6]. The commonest way for spermatozoa cryopreservation is conventional slow freezing and the ideal cooling rate is expected neither to be too fast nor too slow, which is actually a fragile balance to keep.

In theory, the rate of cooling/warming and the concentration of cryoprotectants are inversely related. That is to say, the faster cooling/warming rate is undertaken, the lower concentration of cryoprotectant is needed [7]. One way to increase the rate of cooling is to minimize both the sample volume and amount of cryoprotectant solution, which means a very large surface area-to-volume ratio. Moreover, the use of advanced and specific devices and materials as supports can markedly increase the rate of cooling.

The present study proposes a new methodology for ultra-rapid cryoprotectant-free cryopreservation of small amounts of human spermatozoa, using a PDMS chip with 10 µm height channel, and leads to a satisfactory cryopreservation effect. To find the best parameter of channel for cryopreservation of spermatozoa, we design channels with a height of 10 µm, 50 µm, 100 µm separately (Figure 1a, 1b, 1c). We found that spermatozoa cryopreserved in channel with a height of 10 µm showed a higher viability and motility compared with the other two groups. If the fluid space around spermatozoa in channel is overmuch, it would be hard to achieve a ideal cooling rate with the increasing sample volume; if the channel space is too narrow, it would be very easy to be obstructed when spermatozoa passes by. The size of spermatozoa head is estimated to be 6 µm, so the spermatozoa just advances in the 10 µm height channel one by one (Figure 1a) and the channel won't be too narrow to be obstructed at the same time.

When compared with conventional freezing, on-chip cryopreservation in 10 µm height channel just displayed a similar viability and motility of frozen-thawed spermatozoa. However, our design has some advantages over conventional freezing in application. First, the fluid volume is small and precise. The volume of the 10 µm height channel is minimized about 5×10^−3^ µL, which is the smallest and most precise volume calculated ever. This means a very large surface area-to-volume ratio, which ensures a very fast and mean cooling/warm rate. Second, no cryoprotectant is needed for our method. Human spermatozoa are too sensitive to tolerate the high concentrations of cryoprotectant conventionally used. Thus high concentrations of permeable cryoprotectants (30%–50%) with the consequent cytotoxic effects due to osmotic stress cannot be applied to spermatozoa cryopreservation. On one hand, our method can avoid the toxicity cryoprotectant caused to cells and decrease the following risk such as abortion and pregnancy loss. On the other hand, our method can simplify the thawing procedure and avoid removing cryoprotectant from spermatozoa. It's reported that removal of cryoprotectant in multiple steps could lead to a loss of spermatozoa motility and membrane disruption to different extents [8]. Third, PDMS is well biocompatible and safe to germ cells. Fourth, PDSM is transparent so that we can get whole control of our operation on spermatozoa with observation under microscope.

Since Nawroth [9] succeeded in cryopreservation of human spermatozoa by direct plunging into liquid nitrogen without cryoprotectants for the first time in 2002, many researchers [10], [11] have made a lot of efforts in cryopreservation for spermatozoa in the absence of cryoprotectants. They commonly believe that as long as we can find a carrier with least volumes of the samples and largest surface area-to-volume ratios, we can realize the cryoprotectant-free cryopreservation of spermatozoa by an ultra-rapid cooling rate. A series of specially designed carriers [11]–[13] have been developed till now, such as human or mouse empty zona pellucidae, cryoloop and the cryotop. But these carriers have their own limitations. Micromanipulation is required for zona pellucidae evacuation and spermatozoa insertion into ZP using human or mouse empty zona pellucidae for cryopreservation [12], [14]. Moreover, as the exogenous protein, mouse zona pellucidae may cause pollution to frozen human spermatozoa. For cryoloop cryopreservation [15], a big minus is that we need to be very careful with the thin and fragile liquid film during whole operation [10]. However, on-chip cryopreservation can overcome these disadvantages above. First, on-chip cryopreservation just loads the fluid without any delicate micromanipulation. Second, there is no need to worry about the exogenous protein pollution in our design. Third, the hand-made PDMS chip is strong enough to store the sample and keep stable during freezing and thawing procedure.

Good-quality spermatozoa DNA is of paramount importance for the correct conveyance of genetic material from one generation to the next, the additional chromosomal damage or strand breaks in DNA caused by freezing and thawing may have detrimental consequences [16]. It is, therefore, crucial to ensure that spermatozoa are cryopreserved in a way as to minimize DNA injury. In the current investigation, our results suggest that the DNA integrity detected by comet assay was similar between pre-frozen and post-thawed spermatozoa no matter by cryopreservation in 10 µm height channel or by the conventional freezing, which means for semen samples of high quality, on-chip cryopreservation and slow freezing were equally effective in terms of recovery of spermatozoa from healthy and fertile population. These results are in accordance with previous studies recruiting such healthy volunteers [17], [18]. However, in contrast to Thomson's study using slow, controlled-rate freezing [19], the amount of DNA damage was increased in the post-thaw spermatozoa samples, suggesting the susceptibility of spermatozoa to damage in the freeze-thaw process, which may due to a specified infertility population. Studies have shown that DNA fragmentation and the percentage of apoptotic spermatozoa significantly increase after cryopreservation [20], possibly owing to formation of reactive oxygen species (ROS), which eventually causes chromatin damage [21].

Another important aspect in the evaluation of semen quality is acrosome integrity. The acrosome is a structure attached to the nucleus of a matured spermatozoa, which contains proteolytic enzymes released to dissolve zona pellucida proteins during fertilization [22]. Acrosome reaction is associated to the activation and exocytotic release of proteolytic enzymes from the acrosome and is thought to play a role in the penetration of the egg investments [23]. FITC-PNA can be used as a reliable probe for detecting acrosome reactions in spermatozoa and the signal representing PNA binding is mainly limited to the acrosomal cap of spermatozoa at the level of fluorescence microscopy. In our study, evaluated by FITC-PNA staining method, frozen-thawed spermatozoa cryopreserved in the 10 µm height channel displayed a higher proportion of intact acrosome compared with that of spermatozoa cryopreserved in the 50 µm or 100 µm height channel, though similar to that of post-thawed spermatozoa by conventional freezing.

Microbial or viral cross-contamination in the liquid nitrogen is a critical issue we are concerned about. However, it is arguable whether the spermatozoa would be infected with bacteria or pathogens mediated by liquid nitrogen, which have leaked into the PDMS chip during storage in a cryotank. According to Endo [24] and Vajta [25], the risk of contamination of germ plasma during cryopreservation and cryobanking in IVF units is small and no report mentions LN2 as a probable vehicle for disease transmission in assisted reproduction treatment. Our study failed to detect the contaminants in the cryotanks, which have been managed in a clean room at 23–28°C with air conditioning for over 5 years of continuous use (data not shown). Further, using an open device, Gimenez et al [26] and Cobo et al [27] have provided the negative evidence of cross-contamination mediated by liquid nitrogen between human pathogens and oocytes/embryos, even from HIV-, HBV-, and HCV-seropositive patients. All in all, information above may suggest that the risk of the cross-contamination between gametes and LN2 in our study is vanishingly small.

In conclusion, this is the first report about successful ultra-rapid on-chip cryopreservation of human spermatozoa without cryoprotectant by direct plunging into liquid nitrogen. Small number of human spermatozoa can be thus successfully cryopreserved without significant loss of important physiological parameters. Ultra-rapid cryopreservation of human spermatozoa without permeable cryoprotectants is a prospective direction for investigations. Anyhow, the final goal of this PDMS chip is to be applied in the cryopreservation of individual spermatozoa. Therefore, in later stage of this project, it is planned to use spermatozoa by TESE as experimental subjects. Another limitation of the micro-device is that we have to spend some time making the chip. All in all, our report of successful recovery of spermatozoa following on-chip cryopreservation is encouraging for further research and clinical implementation.
